# Genome Sequence of *Microbacterium* sp. Strain 3J1, a Highly Desiccation-Tolerant Bacterium That Promotes Plant Growth

**DOI:** 10.1128/genomeA.00713-15

**Published:** 2015-08-27

**Authors:** Maximino Manzanera, Cristina García-Fontana, Juan Ignacio Vílchez, Juan Jesús Narváez-Reinaldo, Jesús González-López

**Affiliations:** Institute for Water Research and Department of Microbiology, University of Granada, Granada, Spain

## Abstract

The genome sequence for *Microbacterium* sp. strain 3J1, a desiccation-tolerant organism isolated from the *Nerium oleander* rhizosphere, is reported here. The genome is estimated to be approximately 3.5 Mb in size, with an average G+C content of 67.7% and a predicted number of protein-coding sequences of 3,310.

## GENOME ANNOUNCEMENT

*Microbacterium* sp. strain 3J1 is a highly desiccation-tolerant Gram-positive bacterium belonging to the *Actinobacteria* phylum and the *Microbacteriaceae* family, and it is isolated from the *Nerium oleander* rhizosphere (1). The genome sequences of other desiccation-tolerant microorganisms have been reported (2–5), including that of the recently described new species, *Arthrobacter siccitolerant* (strain 4J27) (6). In response to changes in osmotic conditions and water activity, these microorganisms produce different compounds (1) known as xeroprotectants (7). These compounds, which are produced to protect essential biomolecules and cell integrity, allow the cell to tolerate extremely low concentrations of water and other chemical insults (8–10), including reactive oxygen species (11). The major water-soluble antioxidants found to date are glutathione (γ-glutamyl-cysteinylglycine [GSH]) and ascorbic acid (12), and the main lipid-soluble antioxidants are tocopherols and carotenes (13), although other antioxidant molecules have been found with important roles in desiccation tolerance (11).

Here, the whole-genome sequence of *Microbacterium* sp. 3J1 is reported based on pyrosequencing technology implemented in the 454 Life Sciences-Roche platform with a combined approach based on 8-kb mate pair and shotgun sequencing (Lifesequencing SL, Valencia, Spain) (14). This technology was used to obtain a total of 109,001 sequences with the mate pair sequencing, rendering an average read length of 286 nucleotides and a total of 128,699 sequences, yielding an average length of 595 nucleotides with the shotgun sequencing strategy. The total number of sequenced bases was 107,758,549, representing a sequencing depth of around 29×. For *de novo* assembly, Newbler Assembler version 2.6 was used, with default parameters. This assembly yielded 30 contigs, of which 15 were >500 bp. The *N*_50_ of the contig assembly was 326,731 bp, and the largest contig was 1,103,902 bp. Mate pair information indicated that most of these contigs were ordered in two scaffolds, the largest comprising 3,402,533 bp. The estimated genome size of 3.5 Mb was deduced from this combination of scaffolds and contigs. Gap-spanning clones and PCR products were used to attempt gap closure, and putative coding sequences were predicted. Genes were annotated with a pipeline implemented at Lifesequencing, and protein-coding sequences (CDSs) were predicted with Glimmer (15–17), RNAmmer (18), tRNAscan (19, 20), and BLAST (21, 22) in combination. Most of the contigs used to obtain complete genomic information for *Microbacterium* sp. 3J1 are contained in two scaffolds, with an average G+C content of 67.7%. The genome was found to contain 3,310 protein-coding genes, 4 rRNA operons, and 44 tRNA genes.

On the basis of this genome sequence, we propose the presence of pathways for the biosynthesis of antioxidants, including glutathione, ascorbic acid, tocopherols, and α-, β-, δ-, ε-, γ-, and ζ-carotene, among many others.

The complete genome sequence of *Microbacterium* sp. 3J1 will contribute to the development of biotechnological applications in the field of anhydrobiotic engineering (23).

### Nucleotide sequence accession numbers.

The complete genome sequence of *Microbacterium* sp. 3J1 has been deposited in the TBL/EMBL/GenBank databases under the BioProject number PRJEB8445 and accession numbers CDWI01000001 to CDWI01000030.
